# Neurosurgery to restore function in cerebral palsy: current practice and emerging therapies

**DOI:** 10.3389/fresc.2026.1789774

**Published:** 2026-04-15

**Authors:** Akshay Sankar, Awa Jobe, Bailey McDonald, Taylor J. Abel, Amit Sinha, Martin G. Piazza

**Affiliations:** 1 School of Medicine, University of Pittsburgh, Pittsburgh, PA, United States; 2 Department of Neurological Surgery, University of Pittsburgh, Pittsburgh, PA, United States; 3 Department of Physical Medicine and Rehabilitation, University of Pittsburgh, Pittsburgh, PA, United States

**Keywords:** cerebral palsy, deep brain stimulation (DBS), dystonia, focused ultrasound (MRgFUS), movement disorders, neuromodulation, selective dorsal rhizotomy (SDR), spinal cord stimulation

## Abstract

Cerebral palsy (CP) affects 1.5–2.5 per 1,000 live births and manifests as diverse movement disorders including spasticity, dystonia, and mixed phenotypes that significantly impact motor function and quality of life. This review examines surgical and neuromodulatory interventions for medically refractory CP-associated movement disorders. Selective dorsal rhizotomy (SDR) offers a novel treatment option for spastic diplegia in ambulatory children (GMFCS II-III), with combined dorsal-ventral rhizotomy showing promise for mixed presentations. Peripheral neurectomies provide targeted focal spasticity management with sustained improvements in muscle tone and walking speed. Intrathecal baclofen (ITB) effectively reduces spasticity and dystonia through programmable drug delivery, though infection rates and potential scoliosis progression require monitoring. Deep brain stimulation (DBS) targeting the globus pallidus internus demonstrates efficacy for dystonia-predominant CP, with younger patients showing greater improvement. Emerging cerebellar DBS approaches show early promise for spasticity and mixed presentations. Spinal cord stimulation (SCS) may benefit select patients with spasticity or painful dystonia, though evidence remains limited. Focused ultrasound represents a novel noninvasive ablative option currently under investigation. Treatment selection requires multidisciplinary evaluation considering movement disorder phenotype, functional goals, patient age, and family factors. As understanding of CP pathophysiology advances, mechanism-based, individualized treatment algorithms will increasingly optimize functional outcomes for this heterogeneous patient population.

## Introduction

Cerebral Palsy (CP) is a group of developmental disorders originating from non-progressive insults in the developing fetal or infant brain, affecting the development of movement and posture (1). CP is one of the most prevalent childhood physical disabilities, affecting approximately 1.5–2.5 per 1,000 live births, with more than 80% attributable to perinatal events (1–3). CP-associated movement disorders include several subtypes: spastic, dystonic, choreoathetotic, dyskinetic, and ataxic manifestations [Table 1, (4)]. Spastic CP accounts for 80% of all cases and manifests as increased muscle tone, exaggerated reflexes, and velocity-dependent stiffness (3, 5–7). Dystonic CP, a subtype of dyskinetic CP affecting 10%–20% of individuals with CP, manifests as increased muscle tone and involuntary contractions (3, 5–7).

**Table 1:** Cerebral palsy (CP) related movement disorders (1–5, 7).

Movement disorder	Pathophysiologic mechanism	Current treatment options
Spasticity	Loss of inhibitory UMN control results in overreactive spinal reflex pathways, which leads to velocity-dependent resistance of the stretch reflex mediated by Ia afferent pathways.	- Botulinum toxin injections - Skeletal muscle relaxants (e.g., dantrolene, tizanidine) - Baclofen (enteral or intrathecal) - Selective dorsal rhizotomy - Phenol injection - Selective neurotomy
Dystonia (dyskinetic CP)	Dysfunction in the motor network involving the basal ganglia, thalamus, cerebral cortex, and cerebellum impairs neural inhibition, leading to hypertonic, hypokinetic postures.	- Baclofen (enteral or intrathecal) - Anticholinergics - Ventral rhizotomy - DBS (typically GPi)
Choreo-athetosis (dyskinetic CP)	Structural dysfunction in parts of the basal ganglia, including the globus pallidus, caudate nucleus, and putamen, causing slow, writhing athetotic movements. Dopaminergic impairment, leading to poor inhibitory feedback resulting in rapid, irregular choreic movements. Together these lead to a hypotonic, hyperkinetic presentation.	- Anticholinergics - Benzodiazepines - Anticonvulsants - Antidopaminergics - Muscle relaxants - Botulinum toxin injections - Physical Rehabilitation
Mixed spasticity-dyskinesia	Dysfunction in both the UMN pathways and the basal ganglia motor network leads to combined overreactive muscle contractions and velocity-dependent stretch reflex resistance.	- Baclofen (enteral or intrathecal) - Anticholinergics - Botulinum toxin injections - Combined rhizotomy
Ataxia	Rarest subtype resulting from perinatal injury to the cerebellum leading to impaired motor control, resulting in impaired balance and motor coordination.	- Current treatment focused on therapy and rehabilitation - Reports of transcranial cerebellar stimulation

UMN, upper motor neuron; DBS, deep brain stimulation; GPi, globus pallidus internus.

Treatment of CP-associated movement disorders is complex due to the frequently mixed nature of presentations, with overlapping movement patterns responding differently to interventions. While techniques such as selective dorsal rhizotomy, peripheral neurectomies, and intrathecal baclofen pump remain effective for specific presentations, targeted neuromodulation strategies including deep brain stimulation and spinal cord stimulation may offer a higher ceiling for improving functional outcomes through precise, adjustable modulation of pathological neural circuits (2, 8). CP movement disorders significantly impact motor function, emotional well-being, and health-related quality of life (HRQoL). Many patients experience chronic pain, sleep disturbances, and challenges with daily activities, highlighting the substantial burden on individuals and their families (3, 5, 9–12).

## Selective neurotomy/neurectomy and selective dorsal rhizotomy

Selective dorsal rhizotomy (SDR) was first described by German neurosurgeon, Otfrid Foerster, in 1908 (13). SDR involves lesioning sensory dorsal nerve rootlets in the lumbosacral spine interrupting aberrant reflex arcs that contribute to spasticity in CP (13–18). A common approach uses lumbar laminectomy coupled with electromyography to divide 60%–80% of fascicles in abnormal rootlets (18–20). A meta-analysis of three clinical trials comparing SDR with physical therapy (PT) to PT alone demonstrated effective reduction in spasticity and a small improvement in gross motor function, though the latter may not reflect real world data (21–24). Later studies showed improvement in mobility and improved overall QoL, with sustained lower limb spasticity improvements at one year follow-up (15, 17, 20, 25). SDR is most commonly performed in children with spastic diplegic CP who are between 4 and 7 years of age, Gross Motor Function Classification Score (GMFCS) of II or III, and can follow intensive physical therapy post-surgery; however, across centers globally there is significant variability in additional inclusion and exclusion criteria (16, 26). Palliative SDR may be considered in cases of severe spasticity to reduce discomfort and improve positioning, though coordinated movements cannot be restored (14, 27). Notably, SDR is primarily used to treat lower, not upper, extremity symptoms, often necessitating other treatments for the upper extremities and/ or dystonia such as intrathecal baclofen (ITB), toxin injections, or more focused rhizotomy techniques (3, 28, 29).

The role of SDR for managing patients with co-existing dystonia is debated. Since spasticity is velocity-dependent while dystonia involves involuntary, sustained muscle contractions, disrupting spinal reflex arcs through SDR can unmask underlying dystonia. Because of this, many recommend avoiding SDR in the presence of dystonia due to underlying dystonia possibly becoming more prominent after spasticity is relieved, which can then decrease quality of life unless ITB is introduced (28, 30). Several limited studies have suggested that combined dorsal and ventral rhizotomies (CDVR) showed promise in patients with mixed dystonic-spastic CP (27, 31, 32). A case series of 7 children with spastic and dystonic quadriplegia with mean preoperative Modified Ashworth Score (MAS) of 3.8 were treated with CDVR showing significant muscle tone reduction with mean postoperative MAS of 0.4 (33).

Spinal deformity is a significant association to consider, although research has shown mixed results; it is unclear if deformity changes compared to the natural history. A systematic review of 1,485 patients found 31.6% scoliosis incidence after multi-level laminectomy SDR technique, with female sex as a significant risk factor (34). Contemporary surgical approaches to SDR vary with respect to extent of spinal exposure. While multilevel laminectomy has widely been utilized, limited single-level laminectomy techniques have been developed to reduce the extent of posterior element removal with the goal of decreasing risk of worsening spinal deformity. Available data suggest comparable functional outcomes, though differences in spinal alignment and long-term musculoskeletal effects continue to be evaluated (35, 36). Other complications include hyperesthesia (14%), superficial wound infection (3.3%), urinary retention (1.3%), and spinal fluid leakage (<1%) (18, 37, 38). A study comparing CDVR with ITB found fewer infections, approximately 15 fewer follow-up visits, and ITB being approximately 4.6 times more expensive than CDVR over 10 years (37–39).

Selective neurectomy/ neurotomy, consists of completely or partially cutting a nerve to reduce overactivity of spastic muscles, providing permanent treatment for limb spasticity in CP (40). A systematic review on selective lower limb neurotomy for focal spasticity using 26 studies and 766 participants reported that 19 studies demonstrated decreased mean MAS scores in treated muscles with statistically significant MAS reduction in 13 studies, and reduced muscle tone in 95% of participants (40). Additionally, 7 of 13 studies reported statistically significant increases in comfortable walking speed with no study reporting decreased walking speed, despite some reports of loss of muscle strength. Selective musculocutaneous neurotomy for fixed upper extremity spasticity has also been reported in the adult literature and may play a role in refractory upper extremity CP related spasticity (41–43).

Complications associated with neurotomy include infection, hematoma, nerve injury, and unintended damage to surrounding structures. Surgical neurotomy carries risk of complex regional pain syndrome as well as deafferentation dysesthesias (40). However, the risk of these complications has not been well described, and further research is needed. Outcomes and safety profiles vary between general and selective approaches, with selective approaches precisely targeting small nerve branches of hyperactive muscles having lower risk profiles. Proper patient selection and anatomic targeting significantly impact long-term outcomes. Despite promising results from observational studies, there have been no large-scale randomized controlled trials for selective neurotomy.

## Intrathecal/intraventricular drug therapy

Baclofen is a GABA-B receptor agonist that reduces excitatory neurotransmitter release from presynaptic neurons and stimulates inhibitory neuronal signals in postsynaptic neurons, ultimately reducing spasticity. Its unpredictable penetration of the blood brain barrier causes baclofen to have a wide array of side effects including lethargy, nausea, and generalized weakness as well as cardiac arrhythmias, hypothermia, seizures, and respiratory depression (44, 45). Intrathecal baclofen (ITB), administered via an implanted pump that delivers medication directly into the spinal fluid, achieves higher local concentrations and can decrease systemic side effects. The implanted pump can be programmed for precise, automated dosing. ITB is FDA-approved for severe spasticity unresponsive to maximum oral baclofen doses or muscle relaxants and has been used effectively for dystonia as well (3, 46–49). Many short-term studies show reduced spasticity, especially in lower limbs, though upper limb effects are less clear; and some show slight increases in gross motor function and HRQoL. Leland Albright's landmark dystonia ITB studies demonstrated that ITB could effectively manage dystonic symptoms in carefully selected CP patients, establishing the foundation for off-label use in dystonia (46, 50). The treatment dosing for dystonic symptoms is variable but often higher than that for spasticity predominant CP (51–54). ITB has been explored for mixed spastic-dystonia movement disorders, with most use in patients with lower limb dystonia and spasticity resulting in improved walking and lower extremity function (52–54). Longer-term studies showed mild improvement in gross motor function and HRQoL (45, 49, 55). For patients with predominantly dystonia, inadequate response to ITB, or an otherwise poor candidate for a lumbar catheter, intraventricular baclofen (IVB) represents an alternative approach. Pioneered by Leland Albright, IVB delivers directly to the cerebral ventricles to target the thalamic relay, thought to be the site of dystonia pathogenesis (56, 57).

A significant concern for children with CP treated with ITB is scoliosis. Some studies suggest that ITB may be associated with worse spinal deformities, but a causal link has not been appropriately established (58). The potential association with accelerated scoliosis highlights the need to further investigate the relationship between scoliosis progression and need for intrathecal baclofen (49, 58). Final scoliosis correction outcomes are not affected by ITB, and complication rates are not affected by the order of the procedures (59, 60).

Complications with ITB are common, reaching approximately 34% in pediatric patients. Primary infection rates range from 0% to 44% across studies, although subfascial implantation reduced infection risk by 12% compared to subcutaneous implantations (61). Abrupt ITB withdrawal can lead to hypermetabolic state with severe rebound spasticity followed by rhabdomyolysis and multi-organ system failure, making patient and caregiver adherence to refill appointments and recognition of withdrawal symptoms critical for long-term safety.

## Deep brain stimulation

Deep brain stimulation (DBS) is a procedure involving targeted electrical stimulation of deep brain structures using stereotactically implanted electrodes connected to an implantable pulse generator (62). A systematic review of 11 studies across 107 patients aged 5 to 26 years found globus pallidus internus (GPi) DBS improved motor symptoms and reduced disability in individuals with CP with up to 56% improvement (63). DBS outcomes vary by dystonia etiology, with dystonia from perinatal insults having notably worse outcomes compared to other etiologies (63). For primary or genetic dystonia not associated with CP, a meta-analysis of 137 patients across 24 studies showed 51.8% improvement in Burke-Fahn-Marsden (BFM) scores, with GPi targeting superior to posterior ventral lateral thalamic nucleus (64). The heterogeneity of outcomes in CP likely reflects the variable patterns and extent of white matter injury, with more diffuse or severe structural damage leading to more widespread network dysfunction less responsive to focal neuromodulation. Although results are mixed based on diagnosis, some younger patients have shown sustained functional gains, suggesting further study of early DBS in pediatric CP populations is critical to understand the potential effect neuromodulation may have on the developing brain (65). DBS provides adjustability to potentially optimize therapeutic effects as symptoms evolve, offering advantages over permanent ablative procedures. Thus, DBS may be a possible long-term solution for patients whose needs may change over time. Moreover, there is precedent for individualized targeting using awake electrophysiologic basal ganglia recording (65). Currently, DBS is considered off-label for children except in primary dystonia patients 7 years or older (66). The American Academy of Cerebral Palsy and Developmental Medicine (AACPDM) recommends DBS for severe generalized dystonia unresponsive to medications.

Although most published data focus on GPi DBS (67) for dystonia predominant CP, small trials have explored cerebellar DBS for spastic CP (68). Traditional GPi DBS has had limited success treating spasticity because spastic lesions are largely structural and spinal-circuit level problems. A small pilot trial of superior cerebellar peduncle DBS in five mixed dystonic-spastic CP patients reported measurable spasticity reductions with statistically significant decreases in mean MAS at 12 months (from 2.9 ± 0.9 to 1.9 ± 0.6, *p* = 0.0454) (69). Chronic cerebellar radiofrequency stimulation across 600 CP patients in the 1980s demonstrated reduced spasticity in 85% of cases (70). Deep anterior cerebellar stimulation in 10 of 13 CP patients with coexisting spasticity and dystonia showed statistically significant spasticity reductions in upper extremities in 8 patients (median MAS from 3 to 1.5, *p* = 0.01) and lower extremities in 7 patients (median MAS from 3 to 1.75, *p* = 0.02) (71). A case series of cerebellar DBS, targeting dentate nucleus and cerebellar outflow tract, in 3 patients with dyskinetic CP with structural basal ganglia damage reported dystonia improvements (BFM reductions 19%–40%) and reduced muscle tightness (68).

These promising cerebellar DBS results have served as foundation for an active N-of-1 clinical trial testing cerebellar DBS with the Medtronic Percept Neurostimulator for patients 7–25 years old with dyskinetic CP with or without comorbid spasticity and clear history of hypoxic brain injury (72). Clinical trial NCT06122675 currently investigates cerebellar DBS utility for children and young adults with dyskinetic/dystonic CP (73). Providers should consider QoL when deciding an optimal DBS target for a patient. This requires a wholistic view by considering a patient's priorities, values, and movement goals along with objective factors like predominant movement disorder (63).

Complication rates vary but have been reported to occur in up to 22.4% of cases: primarily infections and device-related issues (64). DBS can be associated with stimulation effects such as dysarthria and dyskinesia and other adverse effects including mood changes, cognitive deficits, potential increased suicide risk, and infections; however, most adverse effects self-resolve without permanent damage (74).

## Spinal cord neuromodulation

Spinal cord stimulation (SCS) applies electrical current to the posterior spinal cord using multiple leads implanted in the epidural space connected to an IPG (75). The primary goal of SCS is to reduce or interrupt pain and other neural signals to and from the brain. Although the mechanism of action is not completely understood, several theories have been proposed including local neural inhibition, excitation of nearby axons, and changes in neurotransmitter physiology. SCS has been used primarily to treat chronic neuropathic pain syndromes refractory to medical management and other conservative methods. However, prolonged SCS has been observed to modulate neuronal activities of deep brain structures, which may help disorders of basal ganglia dysfunction (76).

Epidural SCS has been proposed as a potential treatment for spasticity in CP (77). A systematic review of 34 studies suggests SCS is a safe and useful tool for spasticity management with symptomatic improvement in up to 78% of patients. Epidural SCS for spasticity from spinal injury had better outcomes compared to cerebral causes, suggesting spasticity from cerebral causes have more complex, widespread neural network dysfunctions and may be less responsive to SCS alone. These observations underscore the importance of appropriate patient selection to maximize therapeutic benefits for spastic CP. However, the authors clearly state the review did not consider the quality of the studies presented. For example, 13 of the studies were evaluated for bias, and none were found to have a low risk of bias (77).

SCS has been explored as a potential dystonia treatment with mixed results. Initial studies of SCS for cervical dystonia in the 1970s showed improvements in head position, comfort, and global disability (74). A series of 129 dystonic patients treated with cervical SCS reported up to 79% having some level of improvement, with 29% having significant improvement in dystonic posture (78). However, a double-blind, crossover trial in 10 dystonic patients failed to show significant objective improvement in dystonia severity or spasm duration. Cervical SCS for dystonia was abandoned in the 1990s in favor of botulinum toxin injections. Recently, burst SCS has shown promise for painful cervical dystonia: a pilot study of burst SCS in four patients with painful cervical dystonia reported sustained and significant improvements in dystonic pain and motor symptoms, with 95.6% pain reduction on visual analog pain (VAS) scores and 82.1% dystonia improvement on Burke–Fahn–Marsden Dystonia Rating Scale (BFMDRS) scores (77).

Although SCS is an established therapy for chronic neuropathic pain, its application for spasticity and dystonia in CP remains largely investigational. Ongoing clinical trials primarily investigate SCS for different pain indications with few trials specifically designed for dystonia or CP-related movement disorders. More trials investigating SCS for spasticity and dystonia in CP and other mixed movement disorders are needed to establish if SCS is a compelling treatment for the spectrum of CP-related movement disorders.

SCS is relatively safe, but approximately 10% of patients experience complications such as infections and hardware malfunction (77). Further research in large patient cohorts with comprehensive protocols is needed to assess SCS utility and safety for spasticity management. More modern SCS paradigms with improved targeting and technology potentially offer more robust therapeutic profiles with fewer side effects. SCS for dystonia has a similar safety profile to SCS for spasticity, with infection and hardware malfunction as the most common complications.

Focused Ultrasound Focused ultrasound (FUS) is a noninvasive technology creating targeted intracranial lesions. Magnetic resonance-guided FUS (MRgFUS) has facilitated real-time guidance to enable precise targeting and monitor temperature increases during sonication (79). MRgFUS focuses ultrasound energy beams precisely on deep brain targets to ablate target tissue, leading to symptom improvement. MRgFUS has received FDA approval for treating essential tremor and is being investigated for other ablative indications in functional neurosurgery, neuro-oncology, and Parkinson's disease (80, 81).

Recently, a clinical trial began at Children's National (NCT06036199) to use FUS to treat patients with dyskinetic cerebral palsy with GPi lesions and evaluate its safety and impact on quality of life, motor development, pain perception, speech, memory, attention, and cognition. Several studies of MRgFUS for dystonia have shown symptomatic improvement of multiple phenotypes (82). Improvements were predominantly observed in focal hand dystonia with most patients undergoing MRgFUS targeting ventrooralis (Vo) thalamus.

Reported complications include transient neurological deficits such as transient facial palsy, balance/gait problems, and long-term sensorimotor side effects including persistent arm pain, dysarthria, and persistent unilateral tongue numbness. Some studies reported new or worsened dystonia after MRgFUS thalamotomy for tremor (82). Little long-term data exists of MRgFUS, necessitating further investigation to better understand its safety and role in treatment of cerebral palsy related movement disorders.

## Conclusion

**Figure 1:**
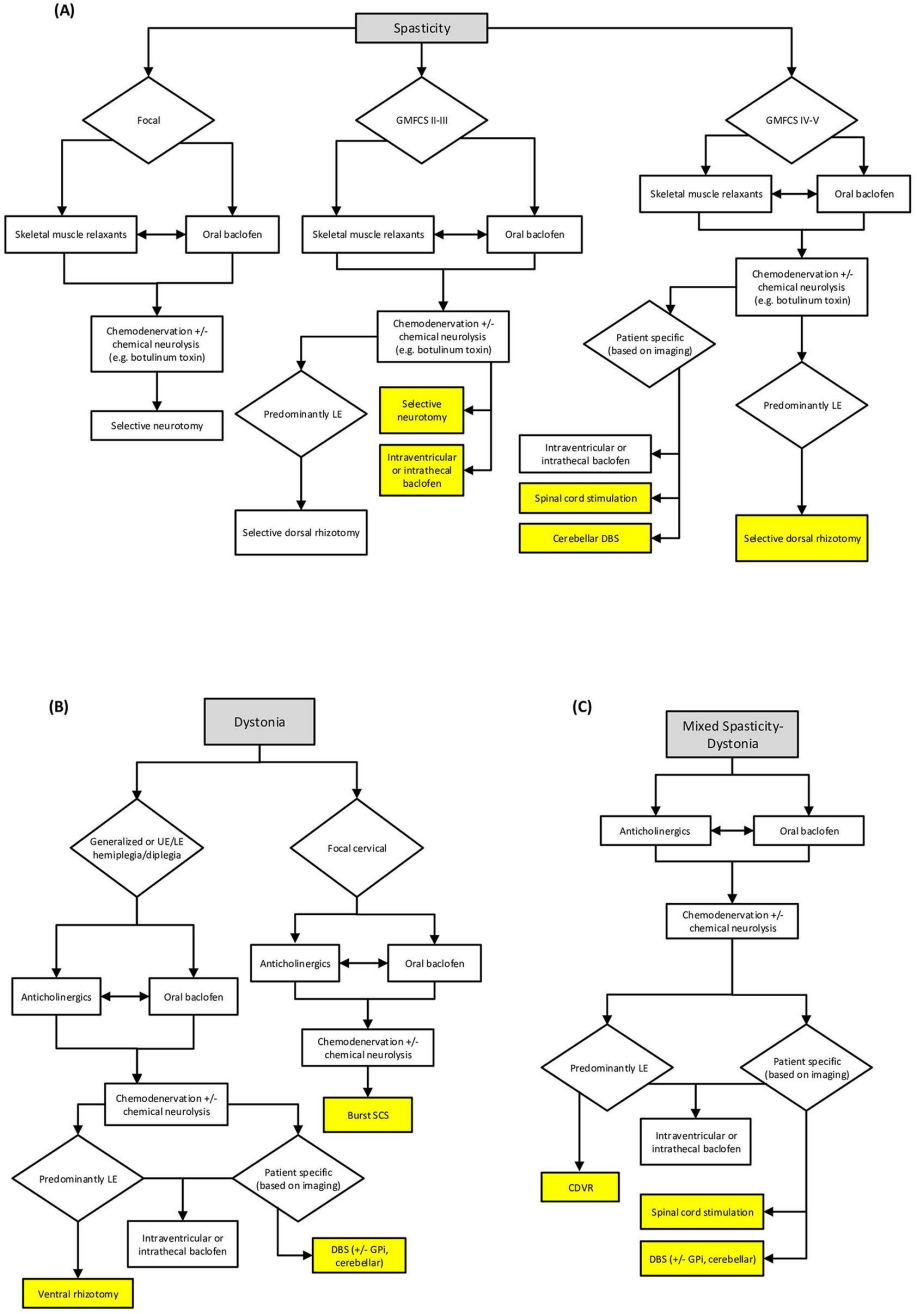
Sample clinical decision algorithm for common movement disorders in cerebral palsy. This depicts a sample decision pathway for children with increasing severity of spasticity **(A)**, dystonia **(B)**, and mixed spasticity-dystonia **(C)** and is not meant to replace individualized treatment plans. Investigational treatments are highlighted. (LE, lower extremity; UE, upper extremity; SCS, spinal cord stimulation; GMFCS, gross motor function classification score; GPi, globus pallidus internus; DBS, deep brain stimulation).

Functional neurosurgical interventions offer valuable treatment options for children with cerebral palsy and medically refractory movement disorders. The expanding armamentarium allows individualized treatment algorithms considering patient age, movement disorder phenotype, functional goals, and family factors. Figure 1 depicts a sample treatment pathway; however, treatments must be individualized in these complex patients. SDR should be strongly considered for pure spastic diplegia in ambulatory children, while combined dorsal-ventral rhizotomy shows promise for mixed dystonic-spastic presentations. Peripheral neurotomies offer targeted focal intervention for specific muscle groups. DBS emerges as a novel option for medically refractory dystonia, with cerebellar DBS showing early promise for spastic and mixed presentations. ITB and IVB provide adjustable pharmacologic management but carry risks of infection and scoliosis progression. SCS may benefit patients with mixed patterns or significant pain components, though evidence remains preliminary. Emerging technologies like focused ultrasound offer noninvasive alternatives warranting further investigation. Success for any strategy requires rigorous patient selection, meticulous surgical technique, comprehensive perioperative care, and intensive rehabilitation. Multidisciplinary teams including neurosurgeons, neurologists, physiatrists, orthopedic surgeons, and therapists optimize outcomes through coordinated evaluation and treatment planning. As understanding of CP pathophysiology and neural circuit function advances, rational intervention selection will increasingly rely on mechanism-based approaches tailored to individual patient phenotypes and needs.
